# Hybrid Use of Negative Pressure Therapy in the Management of Partial Wound Closure After Girdlestone Procedure

**DOI:** 10.7759/cureus.8842

**Published:** 2020-06-26

**Authors:** Erin G Andrade, Laurie Punch

**Affiliations:** 1 Surgery, Washington University, St. Louis, USA; 2 Surgery, Barnes-Jewish Hospital, Washington University, St. Louis, USA

**Keywords:** girdlestone, girdlestone pseudoarthroplasty, septic arthritis, cinpwt, delayed primary closure, closed incision negative pressure wound therapy

## Abstract

In a patient with septic arthritis and pressure ulcers requiring bilateral Girdlestone pseudoarthroplasty, hybrid open and closed incisional negative pressure therapy (ciNPT) is used to manage a closed surgical incision confluent with a large, open wound. Hybrid open and ciNPT facilitates both the healing of the primary closure as well as preparation of the wound bed for skin grafting. ciNPT can be used in partially closed wounds in combination with traditional NPT of the open portion of the wound to allow for more successful closure in wounds under tension.

## Introduction

Negative pressure therapy (NPT) in open wounds has been shown to improve healing by augmenting angiogenesis, reducing edema and tension, and increasing tissue perfusion [1-3]. While most commonly used in open wounds, NPT may also be employed with closed incisions. Closed incisional negative pressure therapy (ciNPT) reduces seroma formation, dehiscence, and in some populations surgical site infections [4-7].

Girdlestone pseudoarthroplasty removes the femoral head and allows access to the acetabulum for debridement and drainage in the case of septic arthritis or chronic osteomyelitis [8-10]. Traditionally, Girdlestone wounds were allowed to heal by secondary intention to allow drainage of the infection; however, we have demonstrated in a prior case series the efficacy of NPT with instillation to allow for delayed primary closure [11]. Due to the nature of the disease requiring Girdlestone pseudoarthroplasty, many patients also have severe tissue loss from deep tissue pressure injury which may prevent adequate skin coverage for complete closure. Although the use of ciNPT in surgical incisions is well established, its use in managing partial wound closure has not been studied. Here we present a patient with septic arthritis and decubitus ulcers requiring bilateral Girdlestone pseudoarthroplasty; hybrid ciNPT applied over a closed surgical incision that is confluent with a large, open wound is used to facilitate both healing of primary closure as well as preparation of the wound bed for skin grafting.

## Case presentation

A 27-year-old male presented to an emergency room with fevers and new drainage from pressure ulcers over his sacrum, left hip, and right ischium. He had a history of paraplegia due to a gunshot wound 10 years prior resulting in spinal cord injury at the T11 level. For years, he had suffered from pressure ulcers and had undergone multiple prior local wound debridements. He had a diverting colostomy in place. At the time of his presentation, his labs demonstrated a white blood cell count was 16 x 10^9^/L, hemoglobin 3.5 g/dL, lactic acid 3.5 mmol/L, and albumin 1.2 g/dL. Computed tomography scan demonstrated large ulcers over the sacrum and bilateral buttocks associated with chronic osteomyelitis of the sacrum, right ischium, bilateral proximal femurs and right intertrochanteric hip fracture associated with septic arthritis. He was started empirically on vancomycin, cefepime, and metronidazole.

**Figure 1:**
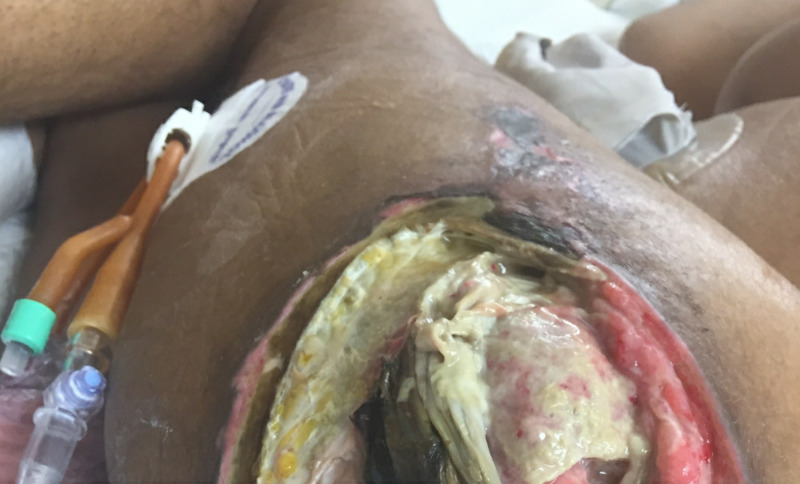
Left Hip Wound Prior to Debridement

**Figure 2:**
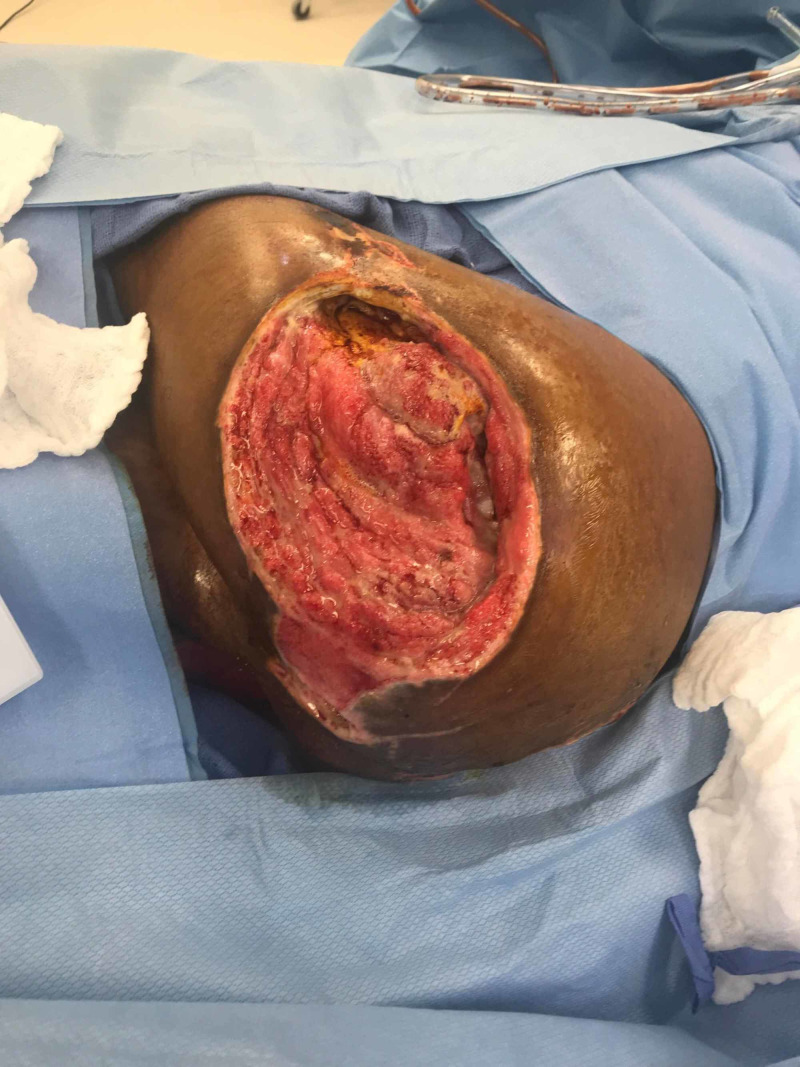
Left Hip Wound After Debridement

Two days after his initial presentation, he was transferred to our facility for complex wound management. On hospital day 1, he underwent excisional debridement of two sites: the confluent sacral and right buttock wounds 9 x 12 cm and the separate left hip wound 17 x 18 cm (Figures 1, 2). NPT with KCI V.A.C. granufoam® was placed. The infectious disease team was consulted and recommended a six-week course of meropenem for Streptococcus pyogenes septicemia and initial wound cultures growing Klebsiella pneumoniae, Pseudomonas aeruginosa and Acinetobacter baumannii.

**Figure 3:**
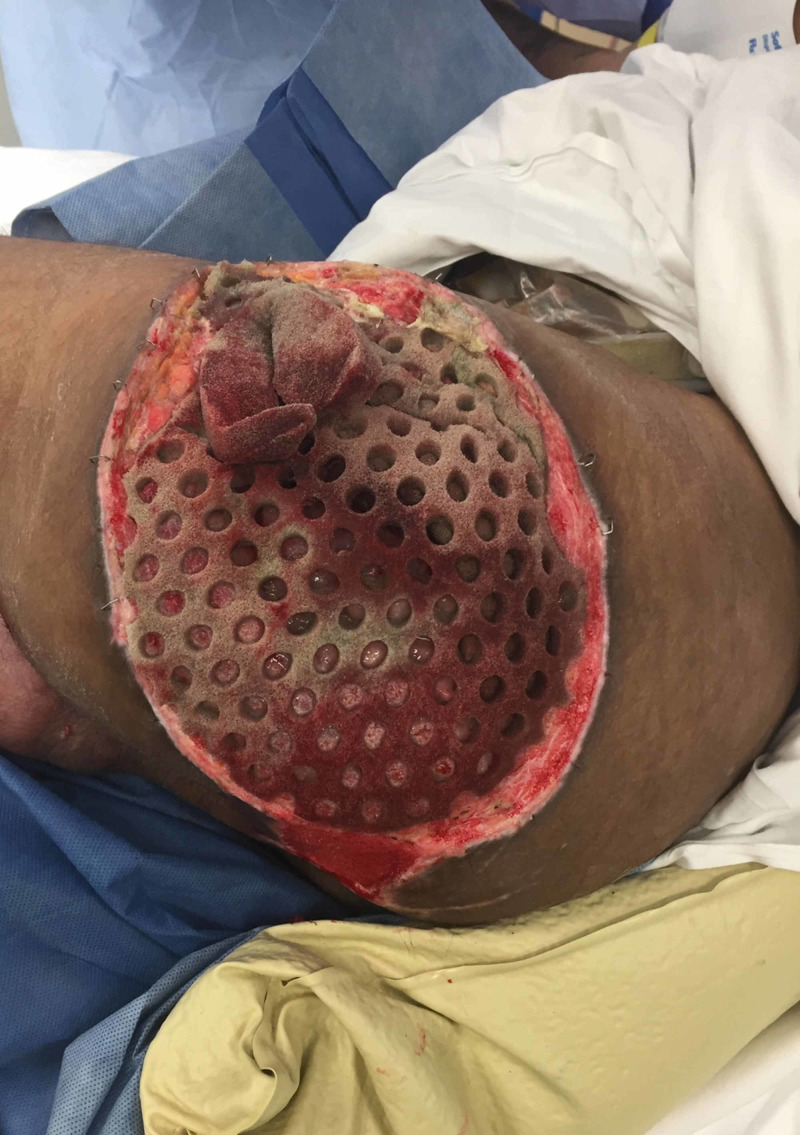
Left Girdlestone Site with Cleanse Choice Sponge

On hospital day 3, he underwent a left Girdlestone procedure using a lateral approach that extended the open wound over his left hip resulting in a 20 x 10 x 10 cm wound that was managed with Promogran Prisma™ to the base and then Cleanse Choice™ sponge (Figure 3). This was placed to standard NPT overnight and then switched to instillation therapy with normal saline the next day.

**Figure 4:**
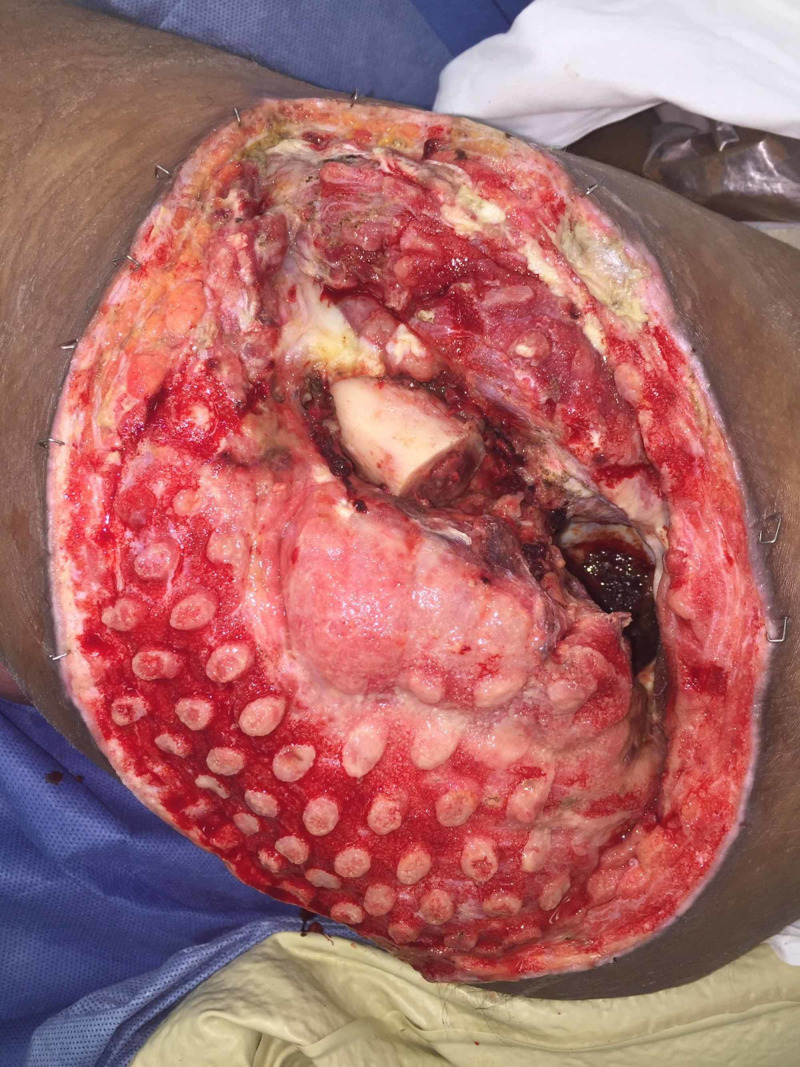
Left Girdlestone Site After Six Days of Instillation Therapy

**Figure 5:**
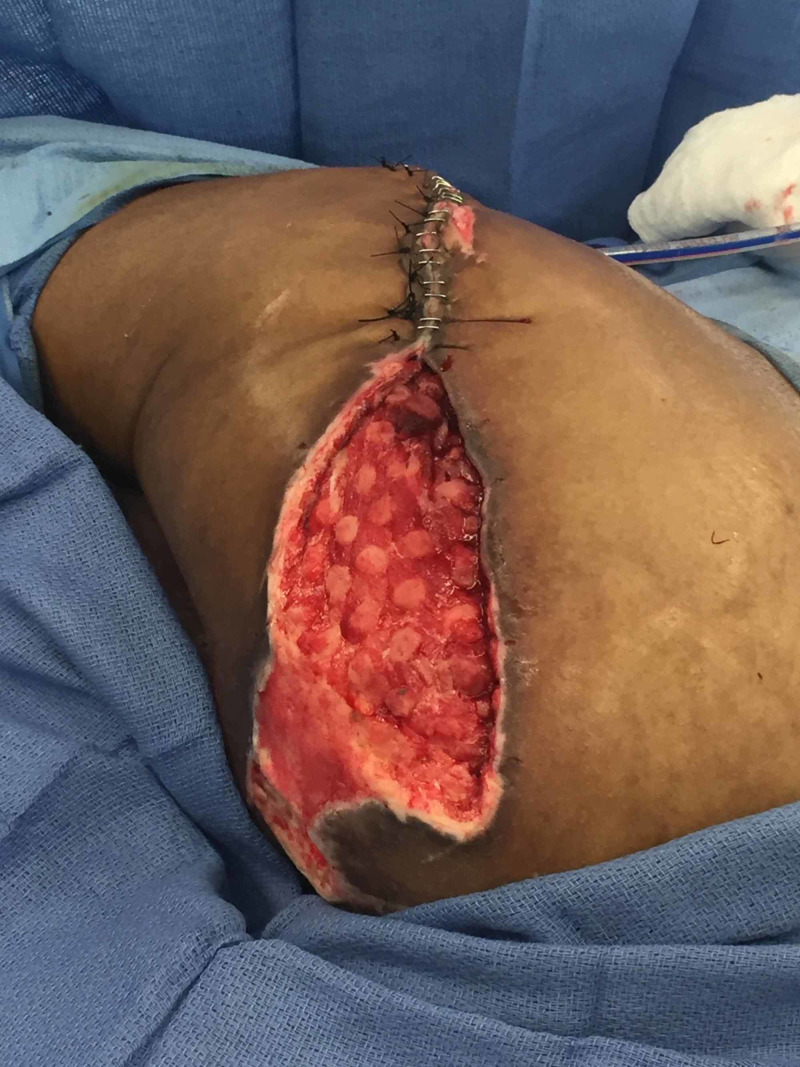
Partial Closure of Left Girdlestone Wound

**Figure 6:**
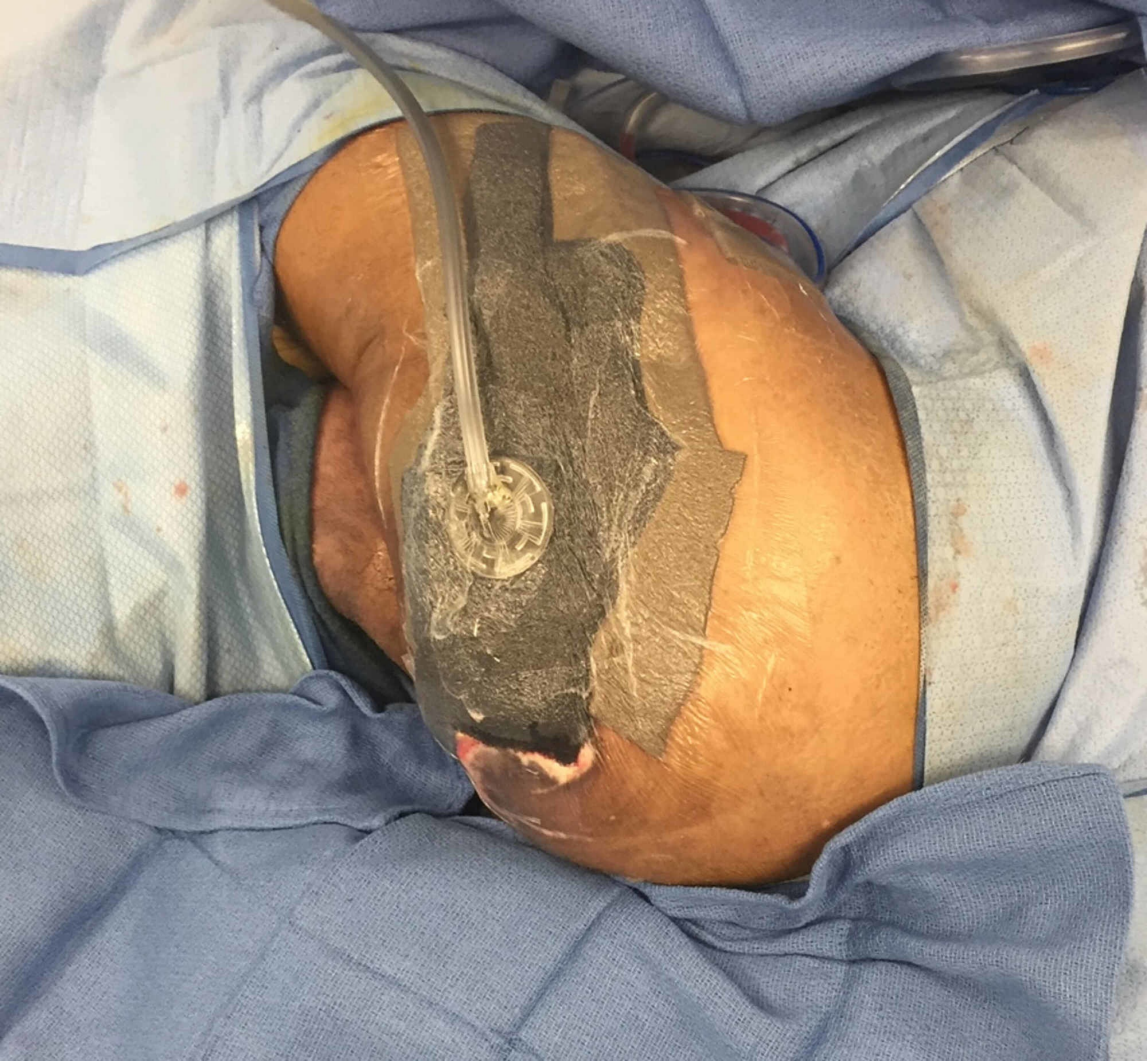
Left Girdlestone Site After Partial Closure with Hybrid Open and Closed Negative Pressure Therapy (NPT)

On hospital day 6, he underwent a right Girdlestone procedure again with a lateral approach. A Cleanse Choice™ sponge was placed for standard NPT overnight and the following day instillation therapy with normal saline was started. During the same operation, his left Girdlestone site was partially closed over 12 cm, which left a 12 x 6 cm area open over the gluteus muscle (Figures 4, 5). Prisma™ was placed to the open wound and PolyMem WIC Silver® was used to cover the contiguous closed incision then black granufoam® was placed over both the open and closed portions (Figure 6). This allowed for one system that was both ciNPT and NPT to prepare the open area for later skin grafting. Right hip tissue cultures grew E. faecalis and left hip bone cultures grew Pseudomonas.

**Figure 7:**
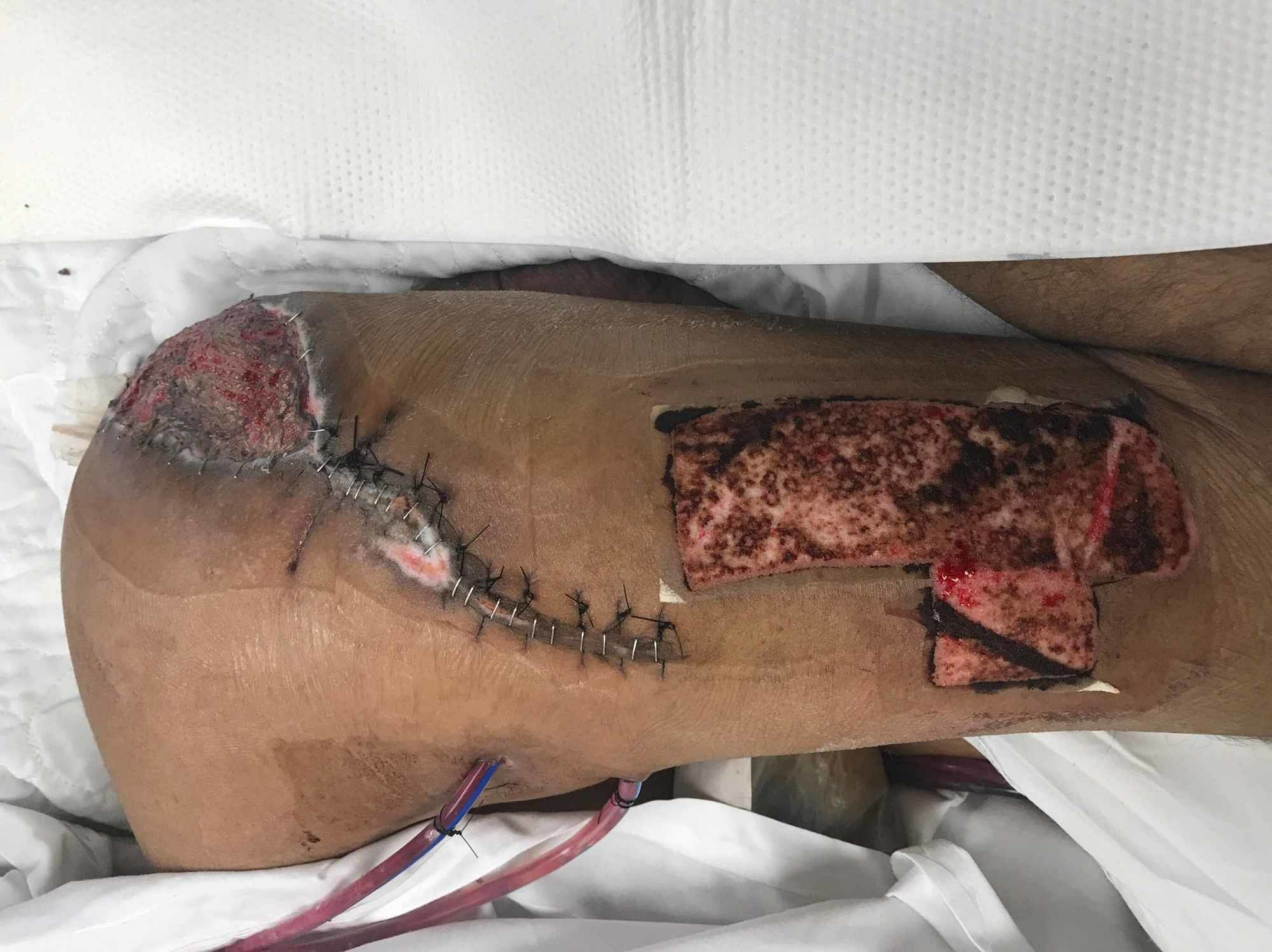
Left Girdlestone Site with Skin Graft Donor Site Distally

On hospital day 11, a split thickness skin graft was harvested from his left thigh and placed over the open wound on his left buttocks excluding the ulcer over his ischium (Figure 7). Adaptic™ was placed over the graft with PolyMem WIC Silver® at the edges and Prisma was placed to the donor site, then black granufoam® was placed over both creating a single NPT system. During the same operation, the right Girdlestone site was closed completely. PolyMem WIC Silver® was placed over the incision and topped with black granufoam® to create ciNPT. Five days later, NPT to the split thickness skin graft and donor site was discontinued. The combination ciNPT and NPT to the left hip was replaced and then taken down on post-operative day 7.

Throughout his hospital stay his nutrition was supplemented with protein shakes at meals and before bed. Frequent turning and an air therapy mattress were utilized to offload pressure and prevent development of new pressure injuries. He was discharged to home on hospital day 19. Antibiotics were transitioned to imipenem with a plan for a six-week course.

**Figure 8:**
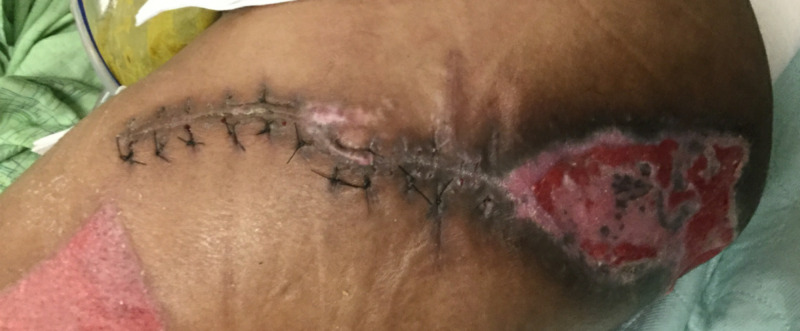
Left Girdlestone Site Four Weeks After Operation

**Figure 9:**
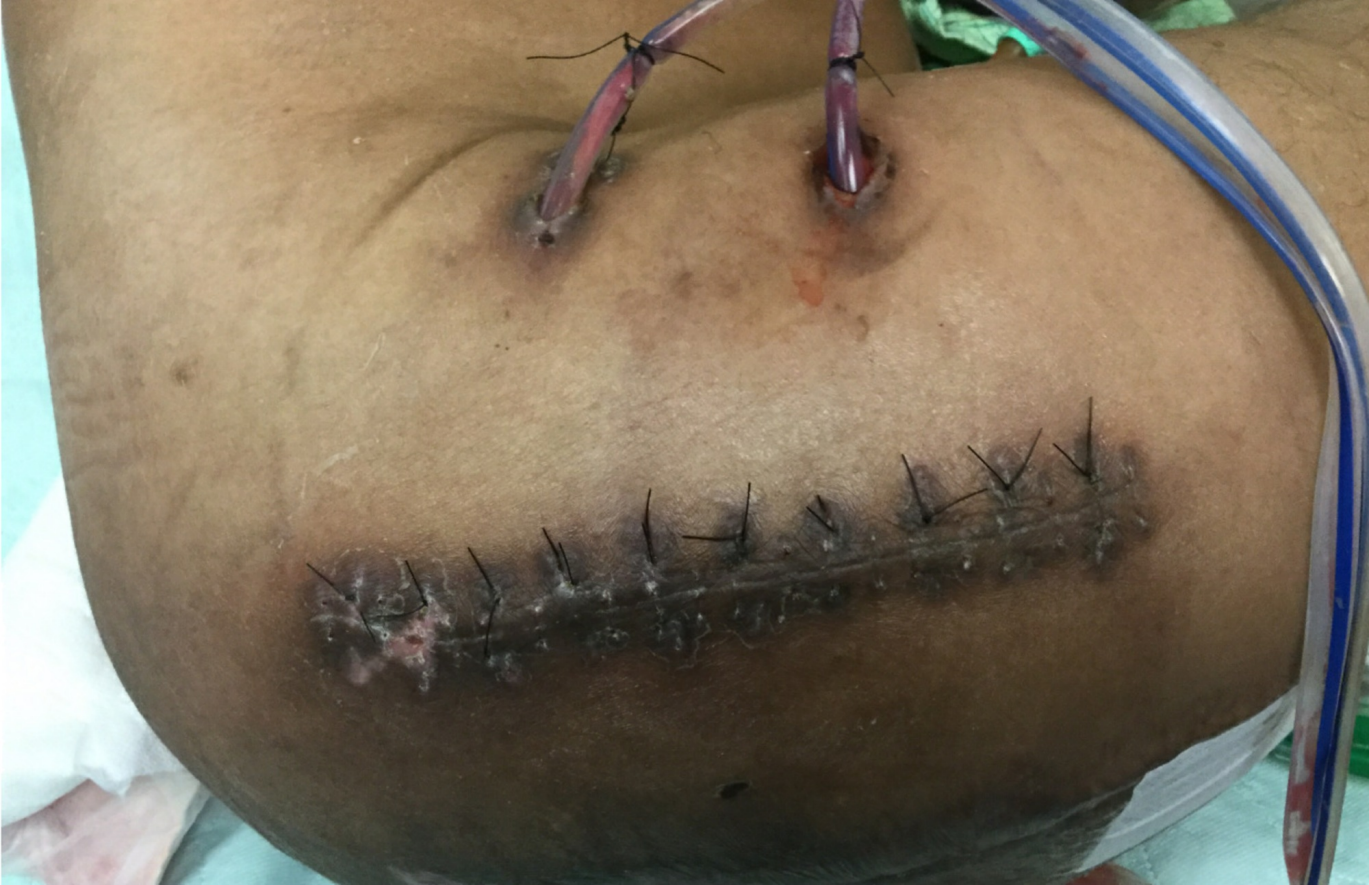
Right Girdlestone Site at Four Weeks After Operation

The patient returned to the emergency room eight days later with a urinary tract infection. He had been unable to complete his intravenous antibiotic therapy at home. He was readmitted for two days to facilitate admission to a skilled nursing facility to aid with intravenous antibiotic administration and wound care. At follow-up four weeks after his final procedure, both operative incisions and his skin graft donor site were healing well and his remaining ischial ulcer had decreased in size (Figures 8, 9).

## Discussion

In this case, we demonstrate multiple applications of NPT: traditional NPT for an open wound with and without instillation, NPT over a split thickness skin graft, NPT over a donor skin graft site, and ciNPT over a completely closed and a partially closed wound. The use of hybrid open and ciNPT permits early partial closure of wounds under tension. Utilizing a combination of custom fit foam for contact over the closed incision and edges of the wound allows for administration of negative pressure across the whole wound while maintaining a seal. ciNPT benefits the closed incision through reduced tension and decreased risk of seroma formation [12,13]. A meta-analysis of ciNPT for closed incisions following orthopedic trauma surgery indicates lower incidence of surgical site infection and dehiscence when compared to traditional dressings [14]. In this large, complex wound, retention sutures were used in combination with ciNPT to reduce the tension on the wound edges. ciNPT increases tensile strength and skin perfusion in closed wounds [15]. The ciNPT acts to distribute the tension of the retention suture across the wound bed and thus decreases erosion of retention sutures. DeFazio et al.’s study of complex wounds under tension showed the use of negative pressure wound therapy (NPWT) combined with retention sutures to be effective in reducing wound size and facilitating wound closure [15]. The advantages of using NPT over the remaining open wound instead of wet to dry dressings include promotion of granulation tissue formation, reduction in wound size, and decreased frequency of dressing changes [16,17]. In our clinical experience, as exemplified in this case, the use of hybrid open and ciNPT allows for closure of wounds with significant tension without dehiscence.

## Conclusions

ciNPT may be used in continuity with traditional open NPT in complex reconstructions. This hybrid method may allow for more successful closures in wounds under tension.
